# Next-Generation Sequencing in Sporadic Medullary Thyroid Cancer Patients: Mutation Profile and Disease Aggressiveness

**DOI:** 10.1210/jendso/bvae048

**Published:** 2024-04-24

**Authors:** Aditya S Shirali, Mimi I Hu, Yi-Ju Chiang, Paul H Graham, Sarah B Fisher, Julie Ann Sosa, Nancy Perrier, Spandana Brown, Vijaykumar R Holla, Ramona Dadu, Naifa Busaidy, Steven I Sherman, Maria Cabanillas, Steven G Waguespack, Mark E Zafereo, Elizabeth G Grubbs

**Affiliations:** Department of Surgical Oncology, The University of Texas MD Anderson Cancer Center, Houston, TX 77030, USA; Department of Endocrine Neoplasia and Hormonal Disorders, The University of Texas MD Anderson Cancer Center, Houston, TX 77030, USA; Department of Surgical Oncology, The University of Texas MD Anderson Cancer Center, Houston, TX 77030, USA; Department of Surgical Oncology, The University of Texas MD Anderson Cancer Center, Houston, TX 77030, USA; Department of Surgical Oncology, The University of Texas MD Anderson Cancer Center, Houston, TX 77030, USA; Department of Surgery, University of California-San Francisco (UCSF), San Francisco, CA 94143, USA; Department of Surgical Oncology, The University of Texas MD Anderson Cancer Center, Houston, TX 77030, USA; Department of Endocrinology, Houston Methodist Hospital, Houston, TX 77030, USA; Institute of Personalized Cancer Therapy, The University of Texas MD Anderson Cancer Center, Houston, TX 77030, USA; Department of Endocrine Neoplasia and Hormonal Disorders, The University of Texas MD Anderson Cancer Center, Houston, TX 77030, USA; Department of Endocrine Neoplasia and Hormonal Disorders, The University of Texas MD Anderson Cancer Center, Houston, TX 77030, USA; Department of Endocrine Neoplasia and Hormonal Disorders, The University of Texas MD Anderson Cancer Center, Houston, TX 77030, USA; Department of Endocrine Neoplasia and Hormonal Disorders, The University of Texas MD Anderson Cancer Center, Houston, TX 77030, USA; Department of Endocrine Neoplasia and Hormonal Disorders, The University of Texas MD Anderson Cancer Center, Houston, TX 77030, USA; Department of Head and Neck Surgery, The University of Texas MD Anderson Cancer Center, Houston, TX 77030, USA; Department of Surgical Oncology, The University of Texas MD Anderson Cancer Center, Houston, TX 77030, USA

**Keywords:** molecular testing, next-generation sequencing, clinical outcomes, somatic mutation, chemotherapy, survival

## Abstract

**Context:**

Next-generation sequencing (NGS) analysis of sporadic medullary thyroid carcinoma (sMTC) has led to increased detection of somatic mutations, including *RET* M918T, which has been considered a negative prognostic indicator.

**Objective:**

This study aimed to determine the association between clinicopathologic behavior and somatic mutation identified on clinically motivated NGS.

**Methods:**

In this retrospective cohort study, patients with sMTC who underwent NGS to identify somatic mutations for treatment planning were identified. Clinicopathologic factors, time to distant metastatic disease (DMD), disease-specific survival (DSS), and overall survival (OS) were compared between somatic mutations.

**Results:**

Somatic mutations were identified in 191 sMTC tumors, including *RET* M918T (53.4%), other *RET* codons (10.5%), *RAS* (18.3%), somatic *RET* indels (8.9%), and *RET/RAS* wild-type (WT) status (8.9%). The median age at diagnosis was 50 years (range, 11-83); 46.1% were female. When comparing patients with *RET* M918T, *RET-*Other, and *RET* WT (which included *RAS* and *RET/RAS* WT), there were no differences in sex, TNM category, systemic therapy use, time to DMD, DSS, or OS. On multivariate analysis, older age at diagnosis (HR 1.05, *P* < .001; HR 1.06, *P<* .001) and M1 stage at diagnosis (HR 3.17, *P* = .001; HR 2.98, *P* = .001) were associated with decreased DSS and OS, respectively, but mutation cohort was not. When comparing *RET* M918T to *RET* indels there was no significant difference in time to DMD, DSS, or OS between the groups.

**Conclusion:**

Somatic *RET* mutations do not portend compromised DSS or OS in a cohort of sMTC patients who underwent clinically motivated NGS.

Medullary thyroid carcinoma (MTC) is a rare thyroid malignancy with a 10-year disease-specific survival (DSS) rate of < 30% in patients with advanced disease [1, 2]. Sporadic MTC (sMTC) constitutes approximately 75% of cases, with the prominent driver pathways identified as mutually exclusive mutations in the *RET* proto-oncogene and *RAS* [3-5]. Ciampi et al [5] performed targeted next-generation sequencing (NGS) on tumors from 181 patients with sMTC and found that genetic mutations in *RET* and *RAS* accounted for 55.8% and 24.3% of mutations, respectively. *RET* mutations were associated with a more aggressive phenotype, corroborating the results of previous smaller studies that suggested that *RET* mutations were associated with compromised overall prognosis [5-7].

The use of NGS to inform the clinical management of patients with cancer is expanding into routine oncologic care [8]. The identification of oncogenic alterations in sMTC, especially the *RET* gene, is crucial for targeted therapies with tyrosine kinase inhibitors, such as nonselective multikinase inhibitors (MKIs) or selective *RET* inhibitors, which have shown efficacy in the treatment of sMTC [9-13]. Eighteen percent of sMTCs had no identifiable oncogenic driver on targeted NGS, which may pave the way for using more comprehensive NGS platforms to aid in the identification of other oncogenic targets for targeted therapy [5].

Our current understanding of the landscape of oncogenic drivers in sMTC comes from investigational studies using targeted NGS on tumors from patients with a breadth of clinical presentations, including those with localized disease [4, 5]. However, bolstered by thyroid cancer clinical practice guidelines, most clinicians advocate for NGS use in advanced sMTC cases with progressive locoregional or distant metastatic disease, where NGS findings may be used for therapeutic purposes, henceforth described in this report as clinically motivated NGS [2]. It is unclear whether the mutational profiles of investigational and clinically motivated NGS cohorts are comparable and if the prognostic information from the former applies to the latter cohort with more advanced disease. As such, we sought to identify the genetic landscape of somatic mutations in patients who underwent clinically motivated NGS, which includes a cohort that comprises patients with advanced disease, and to determine whether an association exists between somatic mutation and clinicopathologic behavior. By studying this enriched group of tumors with advanced disease, we add context to the real-world experience of NGS use and interpretation in the clinical setting.

## Methods

### Patient Cohort and Clinical Data

**Figure 1.**
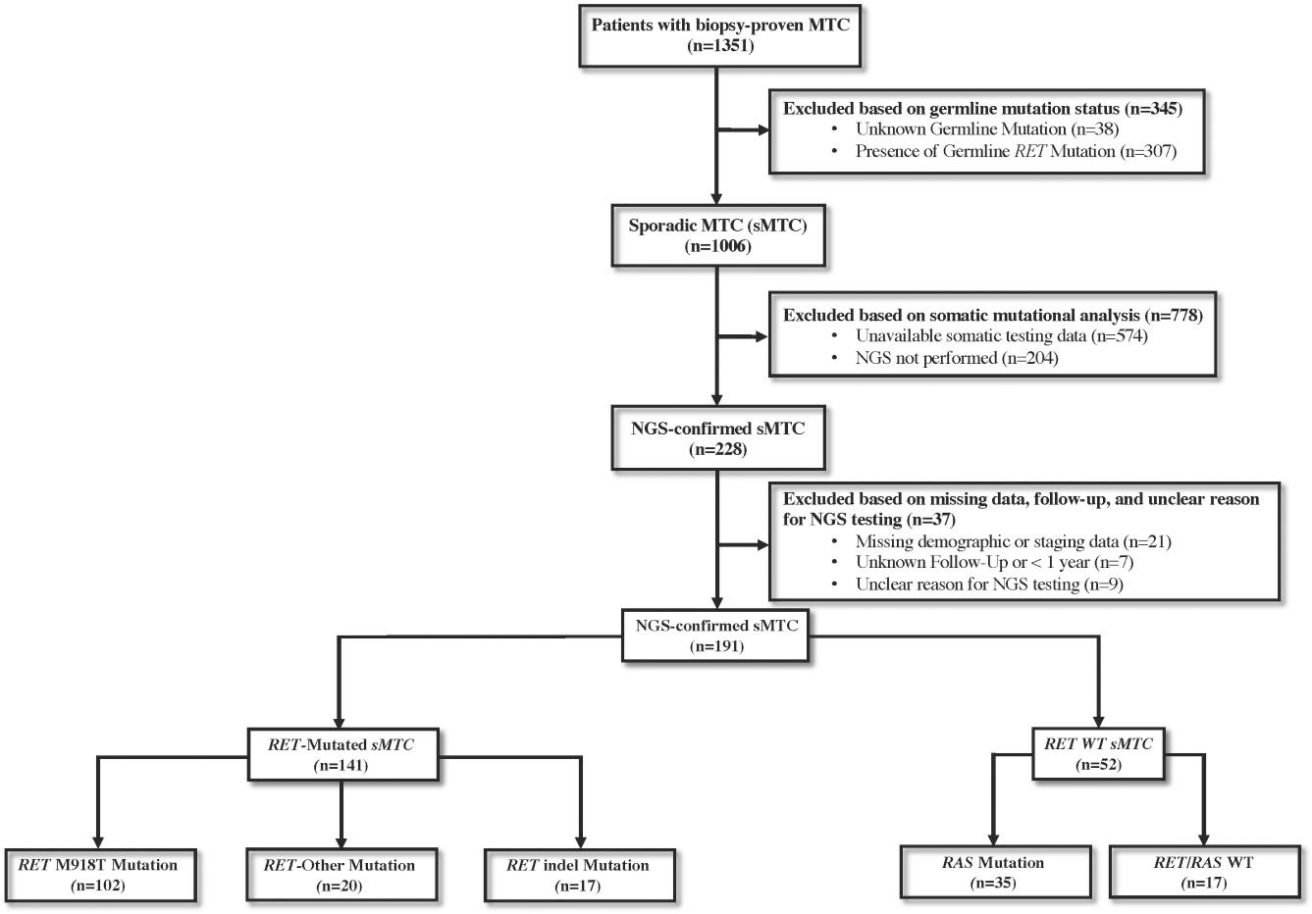
Participant flow diagram of patient selection from the multi-institutional medullary thyroid cancer (MTC) registry used to identify patients with sporadic MTC (sMTC) who underwent next-generation sequencing (NGS).

Following institutional review board approval (IRB# 2021-0234), patients with sMTC who underwent NGS as part of their oncologic care through the study end date of June 31, 2021, were identified from MTC CoRe, a multi-institutional MTC registry. Patients were included if they had a diagnosis of sMTC, defined as pathologically confirmed MTC in the initial biopsy or surgical specimen, and NGS on available tumor tissue performed with clinically motivated intent. Patients were excluded if they had evidence of a pathogenic germline mutation, identified after testing of exons 10, 11, and 13-16 of the *RET* gene or incomplete germline testing, inaccessible NGS results, incomplete demographic or American Joint Committee on Cancer (AJCC) staging information, or follow-up of < 1 year (Fig. 1).

Demographics, clinicopathologic factors, AJCC 7th edition stage at initial diagnosis, NGS-specific data, and systemic therapy use were obtained from the registry and chart review to perform this retrospective cohort study. The primary endpoints were time to distant metastatic disease (DMD), disease-specific survival (DSS), and overall survival (OS) to assess clinical aggressiveness of sMTC. The time of origin was the date of diagnosis, and the events of interest were the date of death due to MTC or last follow-up to determine DSS or date of death due to all causes or last follow-up to determine OS. The time to DMD was defined as the time from diagnosis to the development of any disease outside the locoregional lymph nodes in the neck. Tumor stage at presentation was determined by TNM classification [14]. Patients with DMD within 6 months of initial diagnosis were classified as M1 at diagnosis and excluded from time to DMD analysis. Patients with unknown M status (MX) at diagnosis as a result of absent staging imaging were classified as M0 or M1 if their calcitonin level was < 500 pg/mL or > 500 pg/mL after complete resection of known cervical disease, respectively. This serves as a stringent cutoff for identification of distant metastases as outlined in the 2015 Revised American Thyroid Association Guidelines for the Management of Medullary Thyroid Carcinoma [2, 15].

### Next-Generation Sequencing and Somatic Mutations

Clinically motivated NGS was defined as NGS that was performed for imminent consideration of initiation of systemic therapy, administered in the setting of advanced locoregional or DMD at the discretion of the treating physician. Reasons for clinically motivated NGS testing included initial presentation of aggressive locoregional disease, distant metastases, and elevated biomarkers, or progressive locoregional disease, distant metastasis, and rise in biomarkers without radiographic progression. Some of these individuals did not receive systemic therapy during the timeframe of this study for a number of reasons, including mutational status, patient discretion, comorbidities, and decision for further short-term observation to understand pace of disease growth.

Sequencing was performed using paraffin-embedded or fresh-frozen MTC tumoral tissue from Clinical Laboratory Improvements Amendments–certified molecular diagnostics laboratories utilized in the course of real-world care. Sequencing panels detected mutations (base substitutions, insertions, deletions, copy number alterations, rearrangements, and fusions) but varied in the number of genes detected by the specific panel. Commercially available NGS panels included Tempus, SNaPShot, Oncocomplete^TM^, and FoundationOne^®^. Sequencing at MD Anderson was performed as previously described using 11-, 26-, 46-, 50-, 134-, and 146-gene panels [16-18], ordered at the oncologist's discretion based on what was available at the time of testing. Patients were divided into groups by somatic mutation: *RET* M918T, *RET-*Other [which included non-M918T *RET* mutations (point mutations or substitutions)], *RET* indels (resulted in overall nucleotide deletion), *RAS*, and *RET/RAS* WT (absence of *RET* and *RAS* mutations). *RET* indels were identified on institutional panel testing of ≥ 50 genes. *RET* indels were annotated to determine actionability on the basis of the known or potential functional significance of the mutation, as previously described [19, 20]. The functional role of *RET* indels, as described by the dbSNP (https://www.ncbi.nlm.nih.gov/snp/) and ClinVar (https://www.ncbi.nlm.nih.gov/clinvar) databases, was reported.

### Statistical Analysis

Statistical analysis included the use of Fisher exact or Wilcoxon rank-sum tests where appropriate. Time to DMD, DSS, and OS were evaluated by Kaplan-Meier analysis and Cox proportional hazard models. Factors associated with time to DMD, DSS, and OS were identified by univariate analysis and further evaluated by multivariate analysis if *P* < .20. All multivariate models used a Cox regression mode with the Firth penalized maximum likelihood method for adjusting bias correction. The *RET* indel cohort was not included in logistic regression because of inconsistent assessment of indels across all NGS panels. A subgroup analysis of patients who underwent institutional NGS testing of ≥ 50 genes, which consistently tested for *RET* indels, was performed. All were performed using SAS 9.4 (Cary, NC), with statistical significance defined as *P* < .05 using two-sided testing.

## Results

### Demographic Characteristics

The study cohort included 191 patients with sMTC who underwent NGS (Fig. 1). The median age of this group at diagnosis was 50 years (range, 11-83); 46.0% were female. The median follow-up of these patients was 80.4 months (interquartile range [IQR], 49.6-130.5). The AJCC T category classification at diagnosis was T1 in 32 patients (16.8%), T2 in 38 (19.9%), T3 in 79 (41.4%), T4 in 34 (17.8%), and TX in 8 (4.2%). The AJCC N category at diagnosis was N0 in 15 patients (7.9%), N1a in 17 (8.9%), N1b in 151 (79.1%), and NX in 8 (4.2%). The AJCC M category was M0 in 131 (68.6%) and M1 in 60 (31.4%) after reclassification of 54 (28.3%) patients with MX category based on postoperative calcitonin levels as described in “Methods.” Of the 131 M0 patients, 70 (53.4%) developed distant metastasis by last follow-up. By the last follow-up, 114 patients (59.7%) were initiated on systemic therapy. At last follow-up, 46 patients (24.1%) were deceased.

The median time from diagnosis to NGS testing was 33.3 months (IQR, 9.6-82.4). In the 77 patients who did not receive systemic therapy during the time of this study, NGS was performed for progressive locoregional disease in 19 (25%), progressive distant metastasis in 18 (23%), initial presentation of aggressive locoregional disease in 18 (23%), initial presentation of distant metastases in 10 (13%), progressive rise in biomarkers without radiographic progression in 9 (5%), and initial presentation of elevated biomarkers in 3 (2%).

### Comparison of Somatic Mutations in sMTC

**Figure 2.**
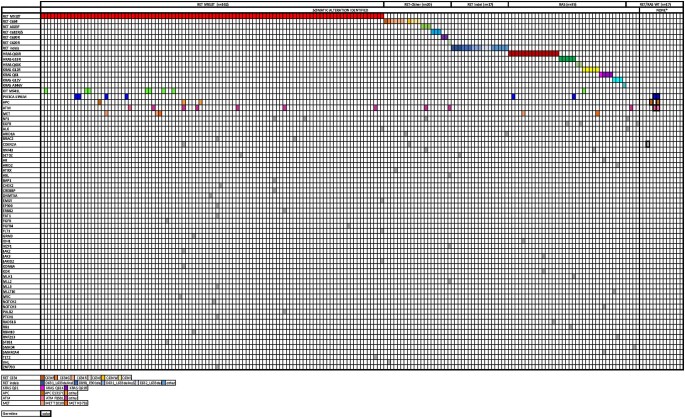
Alteration profile of the 192 sporadic MTC (sMTC) patients who underwent next-generation sequencing (NGS). Each column corresponds to a single case. Genetic alterations are listed on the left. The nonbolded colored squares correspond to the specific somatic alteration, while the bolded colored squares correspond germline mutations.

Of the 191 patients with sMTC, NGS identified somatic *RET* M918T (n = 102 [53.4%]), other non-M918T *RET* codons (*RET-*Other, n = 20 [10.5%]), *RAS* (n = 35 [18.3%]), somatic *RET* indels (n = 17 [8.9%]), and *RET/RAS* WT status (n = 17 [8.9%]). An oncoplot demonstrating these mutations is shown in Fig. 2.

#### *RET* M918T vs *RET*-Other vs No *RET* Activating Mutations

**Figure 3.**
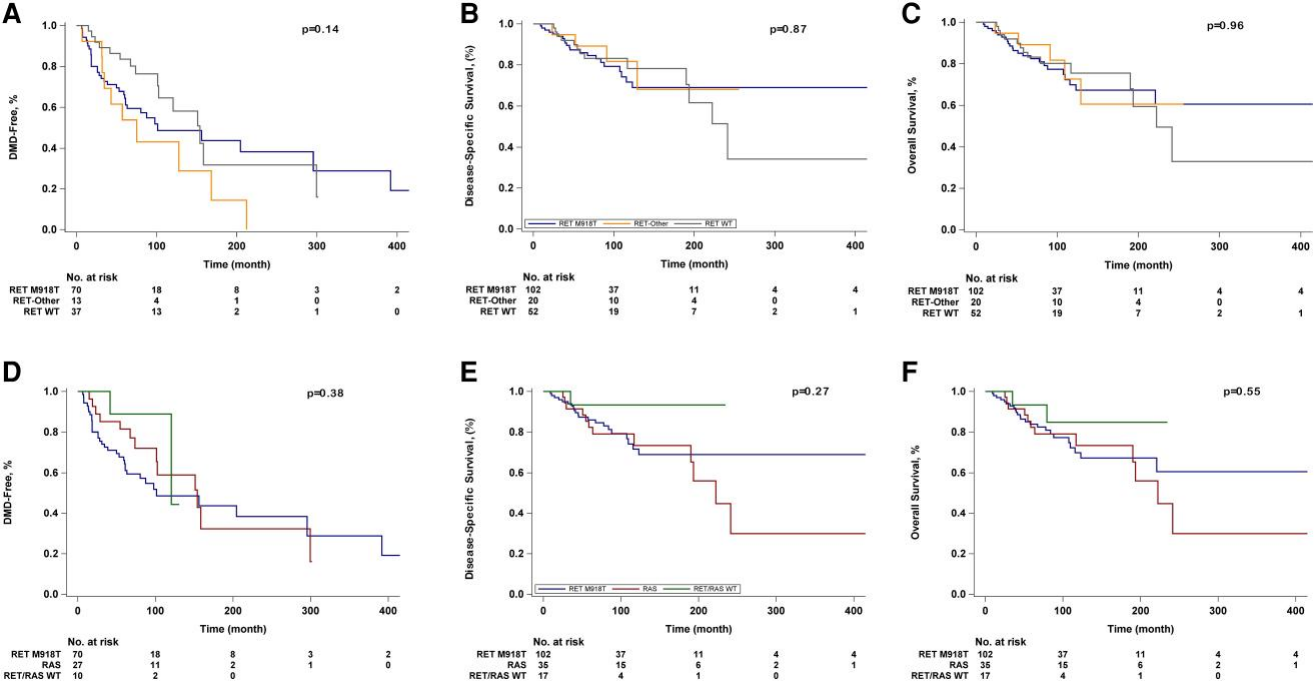
Kaplan-Meier curves of A, time to the development of distant metastasis (DMD), B, disease-specific survival, and C, overall survival among patients with somatic RET M918T, RET-Other, and RET WT status, and D, time to DMD, E, disease-specific survival, and F, overall survival among patients with somatic RET M918T, RAS, and RET/RAS WT status.

Comparisons of clinicopathologic characteristics of patients with *RET* M918T, *RET-*Other, and no *RET* mutations (*RET* WT, which included *RAS+* and *RET/RAS* WT subgroups) are shown in Table 1. There were no significant differences in sex, TNM category, median time from diagnosis to NGS testing, use of systemic therapy, median time from diagnosis to systemic therapy, death on systemic therapy, median time from initiation of systemic therapy to death, or median follow-up among the 3 cohorts. *RET-*Other patients were older at diagnosis than *RET* M918T and *RET* WT patients (median age, 60 years [range, 23–81] vs 50 years [range, 17–83] and 50 years [range, 11–71], *P* = .012). Although there was no difference in M category at diagnosis, of the patients with M0 disease at diagnosis, 37 (52.9%) patients with *RET* M918T, 11 (84.6%) with *RET-*Other, and 16 (43.2%) with *RET* WT developed distant metastases (*P* = .036). There was no significant difference in median time to DMD (Table 1, Fig. 3A, *P* = .14), although the time trended toward being shorter for *RET*-Other than for *RET* WT (75.2 months vs 154.0 months, *P* = .051). There was no significant difference in DSS or OS among the 3 groups. Median DSS was not reached for any of the 3 cohorts. Median OS was only reached for *RET* WT at 242.0 months. The 5-year DSS and OS rates were 85.3% and 83.8% for *RET* M918T, 89.2% and 89.2% for *RET-*Other, and 85.5% and 85.5% for *RET* WT (Table 1, Figs. 3B and 2C, *P* = .87 and *P* = .96, respectively).

**Table 1.** Clinicopathologic characteristics of patients by somatic RET M918T, RET-other, and RET WT Status

	RET M918T (n = 102, A)	RET-Other (n = 20, B)	RET WT (n = 52, C)	*P* value	*P* value (A vs B)	*P* value (A vs C)	*P* value (B vs C)
Age at diagnosis, median (range), y	50 (17–83)	60 (23–81)	50 (11–71)	.012	.003	.38	.027
Female, n (%)	56 (55)	8 (40)	24 (46)	.36	.23	.31	.79
T category at diagnosis, n (%)				.79	.94	.46	.68
T1	20 (20)	4 (20)	5 (10)				
T2	21 (21)	5 (25)	10 (19)				
T3	40 (39)	8 (40)	24 (46)				
T4	15 (15)	3 (15)	11 (21)				
TX	6 (5)	0	2 (4)				
N category at diagnosis, n (%)				.13	.47	.07	.25
N0	7 (7)	0	8 (15)				
N1a	8 (8)	2 (10)	5 (10)				
N1b	85 (83)	17 (85)	35 (67)				
NX	2 (2)	1 (5)	4 (8)				
M category at diagnosis, n (%)				.89	.80	.85	.78
M0	70 (69)	13 (65)	37 (71)				
M1	32 (31)	7 (35)	15 (29)				
Presence of distant metastases after diagnosis, n (%)	37 (53)	11 (85)	16 (43)	.036	.033	.34	.01
Time from diagnosis to NGS testing, median (IQR), mo	28.4 (7.9–67.2)	77.2 (10.0–122.6)	34.2 (16.6–88.7)	.21	.14	.24	.33
Systemic therapy, n (%)*^a^*	65 (64)	13 (65)	25 (48)	.15	.91	.08	.29
Selective RETi	42 (65)	9 (69)	0	<.0001	.75	<.0001	<.0001
Nonselective MKI	39 (60)	8 (62)	24 (96)	.0035	.92	.0006	.012
Other	6 (9)	1 (8)	7 (28)	.054	.86	.041	.22
Time from diagnosis to systemic therapy, median (IQR), mo	48.3 (9.4–88.3)	51.4 (20.3–73.9)	28.8 (14.8–89.9)	.85	.91	.68	.52
Death on systemic therapy, n (%)	20 (31)	5 (38)	9 (36)	.81	.59	.63	.88
Time from systemic therapy to death, median (IQR), mo	33.5 (11.5–45.1)	35.6 (33.7–65.5)	36.0 (18.8–57.0)	.63	.45	.53	.80
Median time to distant metastatic disease, mo (95% CI)	101.4 (61.1–295.4)	75.2 (32.1–168.2)	154.0 (101.4–299.4)	.14	.20	.24	.051
5-year disease-specific survival rate (%)	85.3	89.2	85.5	.87	.74	.91	.72
5-year overall survival rate (%)	83.8	89.2	85.5	.96	.82	.94	.75
Follow-up, median (IQR), mo	80.3 (47.2–126.5)	96.8 (67.8–148.0)	80.1 (58.9–136.7)	.34	.20	.35	.47

The *RET* M918T group includes sMTC patients with a *RET* M918T mutation, the *RET*-Other group includes patients with other non-M918T *RET* mutations, and the *RET* WT group includes patients with *RAS* mutations and *RET/RAS* WT.

Abbreviations: IQR, interquartile range; *MKI*, multikinase inhibitor; NGS, next-generation sequencing; *RET*i, *RET* inhibitor; WT, wild-type.

^*a*^Selective *RET* inhibitors included selpercatinib and pralsetinib. Nonselective multikinase inhibitors included sorafenib, lenvatinib, sunitinib, vandetinib, and cabozantinib. Other systemic therapies included pembrolizumab, tipifarnib, everolimus, and other therapies.

#### *RET* M918T vs *RAS* vs *RET/RAS* WT

After subdividing the *RET* WT cohort into *RAS* (n = 35 [18.3%]) and *RET/RAS* WT status (n = 17 [8.9%]), comparisons of the clinicopathologic characteristics between these cohorts with *RET* M918T mutations (n = 102 [53.4%]) were performed (Table 2). There were no significant differences in age, sex, T and M category, median time from diagnosis to NGS, time from diagnosis to systemic therapy, death on systemic therapy, median time from initiation of systemic therapy to death, or median follow-up. Those with a *RET* M918T mutation were more likely to present with advanced N category and receive systemic therapy than were those with a *RAS* mutation (*P* = .030 and *P* = .014, respectively, Table 2). Of the patients with M0 disease at diagnosis, 37 (52.9%) patients with *RET* M918T, 13 (48.1%) with *RAS*, and 3 (30%) with *RET/RAS* WT developed distant metastases (*P* = .40). There was no significant difference in time to DMD (Fig. 3D), DSS (Fig. 3E), or OS (Fig. 3F) between the 3 groups. Of the 65 patients with *RET* M918T mutations who received systemic therapy, 42 (65%) received selective *RET* inhibitors, and 39 (60%) received nonselective MKIs.

**Table 2.** Clinicopathologic characteristics of patients by somatic RET M918T, RAS, and RET/RAS WT Status

	RET M918T (n = 102, A)	RAS (n = 35, B)	RET/RAS WT (n = 17, C)	*P* value	*P* value (A vs B)	*P* value (A vs C)	*P* value (B vs C)
Age at diagnosis, median (range), y	50 (17–83)	48 (25–78)	55 (11–71)	.51	.69	.27	.42
Female, n (%)	56 (55)	18 (51)	6 (35)	.32	.84	.19	.38
T category at diagnosis, n (%)				.27	.51	.16	.18
T1	20 (20)	3 (9)	2 (12)				
T2	21 (21)	10 (29)	0				
T3	40 (39)	15 (43)	9 (53)				
T4	15 (15)	6 (17)	5 (29)				
TX	6 (5)	1 (2)	1 (6)				
N category at diagnosis, n (%)				.14	.030	.74	.57
N0	7 (7)	7 (20)	1 (6)				
N1a	8 (8)	3 (9)	2 (12)				
N1b	85 (83)	22 (62)	13 (76)				
NX	2 (2)	3 (9)	1 (6)				
M category at diagnosis, n (%)				.38	.34	.42	.17
M0	70 (69)	27 (77)	10 (59)				
M1	32 (31)	8 (23)	7 (41)				
Presence of distant metastases after diagnosis, n (%)	37 (53)	13 (48)	3 (30)	.40	.68	.18	.32
Time from diagnosis to NGS testing, median (IQR), mo	28.4 (7.9–67.2)	35.8 (16.0–107.5)	33.1 (24.3–50.9)	.51	.27	.61	.74
Systemic therapy, n (%)*^a^*	65 (64)	14 (40)	11 (65)	.040	.014	.94	.094
Selective *RET*i	42 (65)	0	0	<.0001	<.0001	<.0001	NA
Nonselective MKI	39 (60)	14 (100)	10 (91)	.0034	.0039	.085	.44
Other	6 (9)	2 (14)	5 (45)	.0068	.57	.0074	.18
Time from diagnosis to systemic therapy, median (IQR), mo	48.3 (9.4–88.3)	27.4 (13.6–118.2)	28.8 (16.9–41.6)	.86	.98	.52	.98
Death on systemic therapy, n (%)	20 (31)	7 (50)	2 (18)	.21	.17	.40	.10
Time from systemic therapy to death, median (IQR), mo	33.5 (11.5–45.1)	36.0 (22.1–57.5)	34.1 (25.9–42.4)	.77	.53	.87	.67
Median time to distant metastatic disease, mo (95% CI)	101.4 (61.1–295.4)	154.4 (73.7–299.4)	120.6 (41.7–150.6)	.38	.46	.43	.72
5-year disease-specific survival rate (%)	85.3	80.0	85.5	.27	.38	.21	.18
5-year overall survival rate (%)	83.8	83.3	93.3	.57	.78	.31	.37
Follow-up, median (IQR), mo	80.3 (47.2–126.5)	76.9 (63.7–172.0)	80.4 (52.4–97.5)	.40	.20	.88	.33

The *RET* M918T group includes sMTC patients with a *RET* M918T mutation, the *RAS* group includes patients with *RAS* mutations, and the *RET/RAS* WT group includes patients with no identifiable *RET* or *RAS* mutations.

Abbreviations: IQR, interquartile range; MKI, multikinase inhibitor; NGS, next-generation sequencing; *RET*i, *RET* inhibitor.

^a^Selective *RET* inhibitors included selpercatinib and pralsetinib. Nonselective multikinase inhibitors included sorafenib, lenvatinib, sunitinib, vandetinib, and cabozantinib. Other systemic therapies included pembrolizumab, tipifarnib, everolimus, and other therapies.

#### Predictors of time to distant metastatic disease, disease-specific survival, and overall survival

Table 3 displays the univariate and multivariate analyses for time to DMD, DSS, and OS. Multivariate analyses of time to DMD could not to be performed as older age at diagnosis (HR, 1.02; 95% CI, 1.00-1.04; *P* = .019) was the only significant covariate on univariate analysis. The results of the univariate analysis for DSS and OS are listed in Table 3. In a multivariate model that included age at diagnosis, M category at diagnosis, systemic therapy use and mutation cohort, older age at diagnosis (HR 1.05; 95% CI, 1.02-1.08; *P* < .001) and M1 category at diagnosis (HR 3.17; 95% CI, 1.59-6.32; *P* = .001) were independently associated with decreased DSS. In a similar multivariate model, older age at diagnosis (HR 1.06; 95% CI, 1.03-1.08; *P* < .001), M1 category at diagnosis (HR 2.98; 95% CI, 1.56-5.69; *P* = .001), and systemic therapy use (HR 2.06; 95% CI, 1.00-4.24; *P* = .049) were independently associated with decreased OS. The mutation cohort was not independently associated with DSS (*RET* M918T HR 4.38, 95% CI 0.58-32.79; *RET-*Other HR 2.15, 95% CI 0.24-19.60; *RAS* HR 6.69, 95% CI 0.86-51.99, *P* = .11) or OS (*RET* M918T HR 1.84, 95% CI 0.49-9.94; *RET-*Other HR 1.62, 95% CI 0.21-8.38; *RAS* HR 2.22, 95% CI 0.49-9.94, *P* = .17).

**Table 3.** Univariate and multivariate analysis of time to distant metastatic disease, disease-specific survival, and overall survival of somatic mutations in sMTC

	Time to distant metastatic disease (n = 120)			Disease-specific survival (n = 174)						Overall survival (n = 174)					
	Univariate			Univariate			Multivariate			Univariate			Multivariate		
	Hazard ratio	95% CI	*P* value	Hazard ratio	95% CI	*P* value	Hazard ratio	95% CI	*P* value	Hazard ratio	95% CI	*P* value	Hazard ratio	95% CI	*P* value
Age	1.02	1.00–1.04	.016	1.05	1.02–1.07	<.001	1.05	1.02–1.08	<.001	1.06	1.03–1.08	<.001	1.06	1.03–1.08	<.001
Female	0.80	0.48–1.34	.39	0.57	0.30–1.08	0.085	—	—	—	0.52	0.28–0.96	.066	—	—	—
T category at diagnosis			.82			.99	—	*—*	*—*			.99	—	*—*	*—*
T1	Reference			Reference						Reference					
T2	0.79	0.34–1.79		0.78	0.28–2.15					0.80	0.31–2.07				
T3	1.00	0.49–2.04		0.98	0.42–2.27					0.97	0.44–2.16				
T4	1.06	0.49–2.04		0.89	0.31–2.58					0.93	0.34–2.50				
N category at diagnosis			.30			.71	—	—	—			.60			
N0	Reference			Reference						Reference					
N1a	1.60	0.45–5.74		0.66	0.12–3.64					0.53	0.10–2.76				
N1b	1.96	0.83–4.61		1.19	0.42–3.38					1.08	0.42–2.76				
M category at diagnosis		—				<.001			.001			<.001			.001
M0				Reference			Reference			Reference			Reference		
M1				3.70	1.92–7.15		3.17	1.59–6.32		3.63	1.95–6.74		2.98	1.56–5.69	
Systemic therapy		—		2.39	1.15–4.96	0.019	2.04	0.96–4.37	.066	2.45	1.22–4.89	.011	2.06	1.00–4.24	0.049
Mutation cohort			.24			.45			.11			.74			.17
RET/RAS WT	Reference			Reference			Reference			Reference			Reference		
RAS	1.86	0.42–8.31		4.42	0.57–34.12		6.69	0.86–51.99		2.22	0.49–9.94		3.43	0.76–15.54	
RET-other	3.72	0.81–17.04		2.59	0.29–23.23		2.15	0.24–19.60		1.62	0.21–8.38		1.26	0.75–15.51	
RET M918T	2.36	0.57–9.89		3.21	0.43–23.93		4.38	0.58–32.79		1.84	0.49–9.94		2.56	0.60–10.92	

M category at diagnosis and use of systemic therapy were not included in the time to distant metastatic disease analysis as patients were all M0 at diagnosis and use of systemic therapy would largely be driven by the development of distant metastatic disease. Patients with *RET* indel were not included in either regression analysis.

#### Somatic *RET* indels

**Figure 4.**
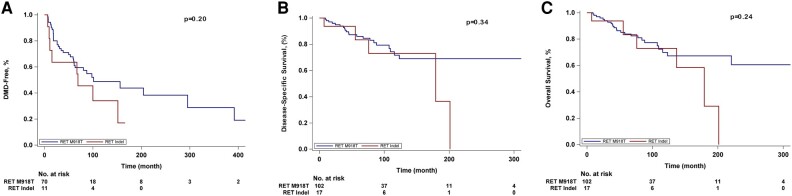
Kaplan-Meier curves of A, time to development of distant metastasis (DMD), B, disease-specific survival (DSS), and C, overall survival (OS) between patients with somatic RET indel and RET M918T.

Seventeen somatic *RET* indels were identified (Fig. 2). Their inferred functional significance in sMTC is characterized in Table 4. Clinicopathologic comparisons of the somatic *RET* indel (n = 17) and *RET* M918T cohorts (n = 102) are described in Table 5. There were no significant differences in median age at diagnosis, TNM category, time from diagnosis to NGS, systemic therapy use, time from diagnosis to systemic therapy use, death on systemic therapy, or median follow-up. Patients with *RET* indels were less likely to be female than were those with *RET* M918T mutations (*P* = .019). There were no significant differences in M category at diagnosis; of the patients with M0 disease at diagnosis, 37 (52.9%) patients with *RET* M918T and 6 (55%) with *RET* indels developed metastases (*P* = .92). There were no significant differences in time to DMD, DSS, or OS (Fig. 4).

**Table 4.** Functional significance and actionability of RET indel (n = 17) based on MD Anderson precision oncology database support

Genetic alteration	Functional significance	Actionable variant	dbSNP	ClinVar	Previous reports
RET c.1894_1899del p.E632_L633del	Activating	Yes: literature-based	rs121913312	Uncertain significance	Elisei et al [21] Dvorakova et al [22] Hong et al [23]
RET c.1891_1898delinsTC p.D631_L633delinsS	Activating: inferred	Yes: inferred	NR	NR	Elisei et al [21]
RET c.1893_1898del p.D631_L633delinsE	Activating: inferred	Yes: inferred	rs121913307	Likely pathogenic	Elisei et al [21]
RET c.2694_2705del p.D898_E901del	Unknown	Potentially	rs121913309	Likely pathogenic	Elisei et al [21]
RET c.1886_1891del p.L629_D631delinsH	Unknown	Yes: literature-based	NR	NR	Elisei et al [21] Hong et al [24] Hong et al [23]
RET c.2698_2710delinsC p.Y900_S904delinsP	Unknown	Potentially	NR	NR	None
RET c.1880_1899delinsCTCAC p.D627_L633delinsAH	Activating: inferred	Yes: inferred	NR	NR	None
RET c.1884_1898del p.L629_L633del	Activating: inferred	Yes: inferred	NR	NR	Elisei et al [21]
RET c.1892_1903del p.D631_R635delinsG	Activating: inferred	Yes: inferred	NR	NR	Subbiah et al [25]

Abbreviation: NR, not reported.

**Table 5.** Clinicopathologic characteristics of patients with somatic RET indels (n = 17) and RET M918T (n = 102)

	RET indel (n = 17)	RET M918T (n = 102)	*P* value
Age at diagnosis, median (range), y	50 (20–77)	50 (17–83)	.56
Female, n (%)	4 (24)	56 (55)	.019
T category at diagnosis, n (%)			.60
T1	3 (18)	20 (20)	
T2	2 (12)	21 (21)	
T3	7 (41)	40 (39)	
T4	5 (29)	15 (15)	
TX	0	6 (5)	
N category at diagnosis, n (%)			.38
N0	0	7 (7)	
N1a	2 (12)	8 (8)	
N1b	14 (82)	85 (83)	
NX	1 (6)	2 (2)	
M category at diagnosis, n (%)			.75
M0	11 (65)	70 (69)	
M1	6 (35)	32 (31)	
Presence of distant metastases after diagnosis, n (%)	6 (55)	37 (53)	.92
Time from diagnosis to NGS, median (IQR), mo	17.7 (43.0–82.3)	28.4 (7.9–67.2)	.73
Systemic therapy, n (%)*^a^*	11 (65)	65 (64)	.44
Selective *RET*i	8 (73)	42 (65)	.43
Nonselective MKI	5 (45)	39 (60)	.36
Other	1 (9)	6 (9)	.12
Time from diagnosis to systemic therapy, median (IQR), mo	15.2 (5.3–24.5)	48.3 (9.4–88.3)	.06
eath on systemic therapy, n (%)	4 (36)	20 (31)	>.99
Time from systemic therapy to death, median (IQR), mo	43.6 (35.6–108.6)	33.5 (11.5–45.1)	.11
Median time to distant metastatic disease, mo (95% CI)	69.0 (9.6–151.5)	101.4 (61.1–295.4)	.20
5-year disease-specific survival rate	83.3	86.0	.34
5-year overall survival rate	83.3	83.8	.24
Follow-up, median (IQR), mo	55.3 (26.7–137.0)	80.3 (47.2–126.5)	.51

The *RET* indel group included sMTC patients with *RET* insertions and deletions, resulting in a net nucleotide deletion, and the *RET* M918T group included sMTC patients with a *RET* M918T mutation.

Abbreviations: IQR, interquartile range; MKI, multikinase inhibitors; NGS, next-generation sequencing; *RET*i, *RET* inhibitors.

^a^Selective *RET* inhibitors included selpercatinib and pralsetinib. Nonselective multikinase inhibitors included sorafenib, lenvatinib, sunitinib, vandetinib, and cabozantinib. Other systemic therapies included pembrolizumab, tipifarnib, everolimus, and other therapies.

## Discussion

Use of NGS platforms for characterizing the molecular profiles of advanced thyroid cancers has moved beyond investigative purposes to allow tailoring of targeted therapies [16]. Whole exomic sequencing and targeted NGS of sMTC identified mutually exclusive mutations in *RET* and *RAS* as the predominant drivers of tumorigenesis in nearly 80% of sMTC and identified novel mutations beyond *RET* M918T, such as other *RET* point mutations and *RET* indels, which are actionable [4, 5, 26]. In the present study, we assessed the association between somatic mutations and clinical behavior in sMTC patients who underwent clinically motivated NGS. This cohort comprised patients with advanced and/or perceived aggressive sMTC; 91% of patients presented with or developed DMD, and 60% received systemic therapy. We found that the cohort of sMTC patients with advanced disease most often harbored somatic *RET* alterations (73%); however, there was no difference in clinical outcomes between patients with somatic *RET* M918T compared with those with *RAS* mutations and *RET/RAS* WT tumors.

Previous reports examining the clinicopathologic characteristics of somatic *RET* mutations in sMTC found that *RET*-mutated sMTC was associated with increased tumor size, nodal and distant metastases, and decreased OS [6, 7]. Ciampi et al [5] performed thyroid-specific NGS on sMTC tumors, irrespective of disease state; 55.8% had *RET* mutations, which were associated with more advanced disease presentation and decreased disease-free survival. Patients with *RET*-driven tumors had decreased OS [5]. In our selective cohort of patients who all possessed clinically aggressive disease behavior, the higher percentage of RET-altered tumors (72%) supports Ciampi et al's findings that harboring such an alteration may be prognostic of a worse diagnosis in all-comers with sMTC. However, when focused only on the aggressive cohort, mutation status loses its prognostic significance.

Our patient cohort included only those who underwent clinically motivated NGS for imminent consideration of systemic therapy, comprising patients with advanced or progressive locoregional or distant metastatic disease. In this group, somatic *RET* mutation status was not independently associated with time to DMD, DSS, or OS. In fact, the patients in the *RET-*Other group were more likely to develop metastases after diagnosis compared with *RET* M918T and *RET* WT patients and had a shorter time to DMD compared with the other 2 cohorts, although not at a statistically significant level. The heterogeneity and small sample size of the *RET-*Other cohort limits our ability to draw discrete conclusions from these results but warrants further investigation with larger studies without inclusion biases. The lack of difference in DSS or OS between somatic *RET* mutations and *RET* WT may be due to the use of selective *RET* inhibitors, which were used in 42% of patients with *RET* mutations in the survival analysis. A subgroup analysis of those who received selective *RET* inhibitors was limited by sample size. These data suggest that in an advanced cohort of patients for whom NGS was performed to guide drug choice, somatic *RET* M918T mutation does not confer worse clinical outcome than other somatic mutations.

Unlike *RET*-mutated sMTC, *RAS*-mutated tumors have been associated with a less aggressive phenotype and better prognosis [5, 27]. In the series by Ciampi et al [5], *RAS*+ tumors represented 24.3% and *RET/RAS* WT tumors represented 5.7% of sMTCs; patients with *RET* WT (*RAS+* and *RET/RAS* WT) tumors had smaller tumors and were less likely to have pathologically positive nodes or DMD at diagnosis compared to *RET*-altered tumors. In our aggressive disease–enriched study population, the *RET* WT cohort of patients consisted of 18.3% *RAS+* and 8.9% *RET/RAS* WT, which was less than one-third of the entire cohort of patients undergoing clinically motivated NGS. The *RET* WT cohort showed no difference in T or M staging at initial diagnosis, time to DMD, DSS, or OS compared to *RET* M918T patients, although they did show less advanced nodal disease at diagnosis than did *RET* M918T tumors. The decreased proportion of *RET* WT identified in our cohort was similarly seen in a previous study of advanced and metastatic sMTC (6.2% *RAS+* and 8.6% *RET/RAS* WT tumors), suggesting that *RET* WT patients are less likely to progress to advanced disease states [28]. While fewer *RAS+* and *RET/RAS WT* tumors developed advanced disease warranting NGS testing than did *RET+* tumors, our data suggest that *RET* WT tumors warranting clinically motivated NGS testing behave similarly to tumors with *RET* M918T mutations. This information is important when counseling advanced patients about prognosis.

While recent NGS studies have shown a *RET* indel prevalence of 14% to 17% in sMTC of various stages [5, 21], our proportion of 9% *RET* indels is in line with what has been identified in advanced sMTC by conventional Sanger sequencing [28]. Seventy-six percent of our *RET* indels resulted in small deletions and insertions in exon 11, which alter the chemical properties and function of the cysteine-rich extracellular domain and potentially activate the RET proto-oncogene [29]. There were no differences in TNM category, use of systemic therapy, time to DMD, DSS, or OS between patients with *RET* indels and those with *RET* M918T mutations. While the interpretation of these data may be limited by statistical power, the lack of difference in survival by mutation status is concordant with what was observed for *RET M918T*, *RAS*, and *RET/RAS* WT.

### Limitations

The limitations of this study include its retrospective design, selection bias to include patients who underwent clinically motivated NGS that were available for review, the possibility that referral bias to tertiary care centers led to improved access to systemic therapies or improved survival, and the small sample size of specific cohorts, which are underrepresented in studies of sporadic MTC. Only patients who underwent NGS for treatment decision making were included in the study, with exclusion of patients with unclear somatic and unavailable NGS data. While this may have biased the results, it also enriched the cohort to one in which targeted NGS was pursued for treatment decision making, rather than investigational or academic purposes, which reflects a real-world clinical practice. Somatic mutations were identified from different and evolving NGS panels over the study period, which is an unavoidable reality of analyzing real-world data. It is unclear to what degree *RET* indels were detectable on commercially available NGS panels. The presence of a concomitant *RET* or *RAS* point mutation and *RET* indel is extremely rare (< 1%) and has never been reported with a *RET* M918T mutation [5]; thus, we included all patients with sMTC who underwent targeted NGS (both institutional and commercial) with the understanding that < 1% may have an unrecognized *RET* indel. Similarly, analysis of pathologic reports of patients over an extended study time period is impacted by different international and society-based pathologic guidelines that makes interpretation of mitoses, necrosis, and/or elevated Ki67 levels challenging. The extended study time prohibits re-evaluation of specimens to address changes in pathologic diagnosis. Multivariate analysis identified systemic therapy use as independently associated with decreased OS, a result that was likely confounded by treatment selection bias and unidentified confounders. A subgroup analysis of patients stratified by type of systemic therapy is limited by small sample size, as this study was not designed to examine the role of systemic therapy on survival. Lastly, this study includes a cohort of patients for whom NGS testing was pursued to guide treatment decision making, and thus underwent NGS testing nearly 3 years after initial diagnosis. As such, we cannot comment on the use of mutation cohort for prognostication in those patients with less advanced disease for whom NGS testing would otherwise not be indicated in a clinical setting.

## Conclusion

Our field has transitioned from utilizing NGS as an investigative tool to one employed in the clinical setting; this study reports novel data from the latter scenario. In an aggressive disease–enriched sMTC group who underwent NGS testing for clinical purposes, while more patients harbored *RET* alterations, we found no difference in clinical outcomes between patients with *RET* M918T mutations, *RAS* mutations, and *RET/RAS* WT. Patients with *RET* indels had no difference in outcomes when compared with those with *RET* M918T mutations, although further investigation is necessary [2]. This is important real-world data to have when counseling patients with aggressive disease; their mutation status does not appear to portend a worse outcome.

## Data Availability

Some or all datasets generated during and/or analyzed during the current study are not publicly available but are available from the corresponding author on reasonable request.

## Abbreviations

AJCC: American Joint Committee on Cancer
DMD: distant metastatic disease
DSS: disease-specific survival
IQR: interquartile range
MKI: multikinase inhibitor
MTC: medullary thyroid carcinoma
NGS: next-generation sequencing
OS: overall survival
sMTC: sporadic medullary thyroid carcinoma
WT: wild-type
